# Tensile and Structural Properties of Antioxidant- and CaCO_3_-Modified Polyethylene Films

**DOI:** 10.3390/polym17162182

**Published:** 2025-08-09

**Authors:** Dmitry Myalenko, Olga Fedotova, Aleksandr Agarkov, Sergey Sirotin, Polina Poletaeva

**Affiliations:** All-Russian Dairy Research Institute (VNIMI), 115093 Moscow, Russia; o_fedotova@vnimi.org (O.F.); a_agarkov@vnimi.org (A.A.); sergey.sirotin@leaneco.ru (S.S.); p_poletaeva@vnimi.org (P.P.)

**Keywords:** modification, dihydroquercetin, calcium carbonate, tensile strength, ATR-FTIR spectroscopy, scanning electron microscopy (SEM), atomic force microscopy (AFM), microplastics

## Abstract

The demand for modified packaging materials increases annually. At the same time, there is growing interest in the development of functional packaging. The incorporation of modifiers, stabilizers, and fillers into polymer matrices can enhance the functionality of the material but may also negatively affect its safety. Polymers are susceptible to degradation, which negatively affects their strength and tensile properties under external factors (physical, chemical or environmental). Packaging containing antimicrobial and antioxidant agents is among the most promising, as it contributes to the product quality during storage. Films based on calcium carbonate (CaCO_3_) and dihydroquercetin (DHQ) remain insufficiently studied, despite their potential. Such materials are especially relevant for fatty products with a large contact surface area, including butter, cheese, and other solid high-fat foods. This study aimed to comprehensively investigate the structural and tensile properties of polyethylene films modified with varying contents of CaCO_3_ and DHQ. The films were produced via blown film extrusion using a laboratory extruder (SJ-28). Surface analysis was performed using scanning electron microscopy (SEM) and atomic force microscopy (AFM). Fourier-transform infrared (FTIR) spectroscopy was used to examine the film’s composition. The results showed that the introduction of more than 40.0 wt.% of CaCO_3_ into the polymer base affected the strength properties. The conducted studies of the physical and mechanical properties of LDPE film samples filled with CaCO_3_ showed significant changes in the samples containing more than 50.0 wt.% of the filler, with an increase in strength of more than 40.0%. The relative elongation at break after 50.0 wt.% decreased by more than 75.0%. These results indicate that to achieve the best strength properties for packaging materials, it is recommended to fill them to a maximum of 40.0 wt.%. The introduction of the antioxidant DHQ had almost no effect on the strength of the modified films. SEM analysis of films with high CaCO_3_ content and DHQ revealed visible antioxidant particles on the film surface, suggesting enhanced antioxidant potential at the interface between the film and dairy products. AFM analysis confirmed that a CaCO_3_ 40.0 wt.% content altered the surface roughness and heterogeneity of the films. FTIR spectroscopy revealed that the incorporation of CaCO_3_ influenced the overall spectral profile of polyethylene, resulting in decreased peak intensities depending on the concentration of the filler. Based on these results, the modified polyethylene-based film with CaCO_3_ and DHQ shows potential for use as food packaging with antioxidant properties.

## 1. Introduction

Polymer-based packaging is widely used across various industries due to its relative ease of production, low cost, light weight, strength and performance characteristics. The main consumers of polymer packaging include manufacturers of electronic, agricultural, food, and pharmaceutical products [1,2].

Among polyolefin-based polymers, polyethylene (PE) is the most commonly used material. It has a high molecular weight structure and exhibits high chemical inertness to various aggressive conditions, which provides significant advantages over many other polymers. There are two main types of PE used in the food industry: low-density polyethylene (LDPE) and high-density polyethylene (HDPE) [3].

Polymers are susceptible to degradation and, under the influence of external factors (physical, chemical, or environmental), can relatively quickly lose their strength, elasticity, and functional properties [3,4]. In modern manufacturing, synthetic polymer materials used for packaging are rarely applied in their pure form; they are typically incorporated with mineral fillers, modifiers, and stabilizers [5]. Due to the wide variety of polymer materials with different structures and properties, it is possible to obtain modified packaging that demonstrates improved and more stable characteristics compared to the base polymer. These materials can also offer targeted functional properties, remain compatible with standard processing equipment and require minimal changes to the production process [6,7,8]. However, it is important to consider that improper selection of modifiers, their concentrations, or processing conditions may compromise the safety of the final material [9,10]. Intermolecular interactions between the synthetic polymer matrix and inorganic fillers can affect the material’s properties at the microscopic level [5].

Various mineral fillers are widely used to modify polymer films, including calcium carbonate (CaCO_3_) [11,12,13,14], clay [15], talc [16], silicon dioxide (SiO_2_) [17], and others.

Global consumption of calcium carbonate (chalk) in the polymer industry is estimated to exceed 10 million tons per year [18]. The addition of CaCO_3_ [19,20] as a filler for LDPE improves impact strength, heat resistance, elastic modulus, tensile strength, and resistance to environmental stress cracking. However, increasing the chalk content leads to a higher degree of crystallinity, which is subsequently associated with a reduction in the elastic modulus.

In the food and dairy industries, a classic example of modified packaging materials is PE film with fillers. This material is used for manufacturing milk and fermented dairy beverage pouches, as well as sheet materials for thermoformed packaging. Dispersed titanium dioxide (TiO_2_) and food-grade carbon black (C) are commonly used as base fillers in such films [21,22].

In the dairy and food industries, CaCO_3_ is the most widely used filler, which has a number of significant advantages: reducing the total cost of a part of the polymer material by introducing a relatively inexpensive filler without compromising product quality. The introduction of CaCO_3_ increases the rigidity, tensile strength and toughness of plastic products, and its presence improves the dimensional stability of plastic products, preventing shrinkage and warping during the manufacturing process. In addition, the addition of CaCO_3_ increases the heat resistance and thermal conductivity of plastics, making them suitable for high temperatures. The particle size of CaCO_3_ can reach 3–7 microns, making it suitable for use as a filler in the production of thin films, which is particularly important in the food industry.

A promising area of research is the development of modified functional packaging, known as “active packaging”, which has a beneficial effect upon contact with food products. Active packaging includes antimicrobial and antioxidant films that incorporate natural plant-based extracts as functional agents [23,24,25,26,27]. These substances migrate from the packaging material to the product over time and prevent surface spoilage by inhibiting undesirable microorganisms [28,29] or slowing oxidative processes. Dairy products, particularly those with a short shelf-life, are especially prone to microbiological contamination and spoilage caused by oxidation. The use of active packaging materials is most appropriate for solid products with a large contact surface area, such as butter, cheese, high-fat products, functional foods, and infant foods [24,28,30,31].

Oxidation is a complex process involving multiple mechanisms, sources, and degradation targets [32]. There are several approaches that can be taken to minimize the risk of oxidative processes, including regulating storage conditions, pre-treating the additives with oxidizing agents, using special packaging, and adding antioxidants [33,34]. The use of natural antioxidants for these purposes appears to be a promising approach. Flavonoids are among the most promising antioxidants, and many of them are widely studied due to their potential health benefits [35,36]. Among them, dihydroquercetin (DHQ) is one of the most promising for several reasons. Firstly, it is a natural polyphenol with a high safety profile, favorable biopharmaceutical properties, and a wide range of biological activities [37,38,39]. DHQ has high antioxidant activity and is as effective as well-known powerful antioxidants such as α-tocopherol, vitamin E, and β-carotene [40]. DHQ, which is used in dietary supplements and medications, has a beneficial effect on numerous physiological processes and inhibits the development of allergic reactions, arthritis, atherosclerosis, cancer, etc. Another important point is that this flavonoid is produced in large quantities, so there is potential to meet the industrial needs of pharmaceutical, cosmetic, and food technologies [41].

Previous studies [42,43,44,45] have examined the interactions between polymer molecules and modifiers in the melt, as well as their influence on the overall set of tensile and sensory properties.

However, the incorporation of various functional components into the polymer matrix of food packaging may present challenges due to differences in operating temperatures or pose risks to quality and safety [46]. Milk is a complex, multi-component system that can be affected by certain packaging modifiers, with the potential to form toxic compounds.

An overview of Russian and global research [6,7,8,42,43,44,45] on synthetic polymer materials with high mineral filler content, including those used in food packaging, highlights the relevance of studying the tensile properties of PE films with varying levels of CaCO_3_ filler. This is also supported by the global trend toward expanding the use of modified and functional packaging. Analysis of the changes in tensile properties and structure of such materials may provide insights into their resistance to degradation. These findings may also help mitigate the risk of microplastic formation and subsequent migration into food products during storage. This study aims to investigate the tensile, structural, and hygienic properties of PE films modified with varying CaCO_3_ and DHQ (natural antioxidant) contents, for potential application in food packaging.

## 2. Materials and Methods

### 2.1. Calcium Carbonate

Calcium carbonate (CaCO_3_), commonly known as chalk, is the most widely used filler for polyolefins [47]. It is typically derived from natural sources such as marble and limestone, and its abundance makes it a cost-effective additive. Chalk-based fillers can increase processing efficiency by reducing cooling rates during molding, raise the operating temperature of the material, and enhance insulation in electrical applications [48]. Additionally, the incorporation of CaCO_3_ enhances the whiteness of opaque or white materials and imparts brightness and gloss to colored surfaces [49].

CaCO_3_ is also extensively used as a filler in the packaging industry. It is an economical additive capable of improving various polymer properties (tensile strength, barrier properties, and impact resistance). When incorporated into polymer films, CaCO_3_ increases rigidity and reduces the permeability to gases and liquids [48,49,50]. CaCO_3_ can also enhance the surface hardness and abrasion resistance of polymer materials [51]. Additionally, it may improve the heat resistance of polymers and reduce the flammability of polymer films. The incorporation of CaCO_3_ can also affect polymer processing behavior, influencing parameters such as melt viscosity and crystallization kinetics [52]. The particle size and shape of the filler are likewise known to impact the final properties of the polymer material [53]. In this study, CaCO_3_ was incorporated using a masterbatch, the technical specifications of which are presented in Table 1.

The particle size distribution of the mineral filler affects the visual appearance of the final product by preventing the formation of visible CaCO_3_ agglomerates on the surface. In addition, particle size influences the material’s ability to form joints. A uniform composition of the chalk-based filler ensures consistent thermal conductivity, which facilitates the formation of smooth and strong joints.

### 2.2. Dihydroquercetin

Dihydroquercetin (DHQ) is a natural antioxidant and polyphenol primarily extracted from the root wood of Dahurian larch (*Larix gmelinii*) and certain other coniferous species. It can also be obtained in smaller amounts from the seeds of milk thistle and peony. DHQ is a flavonoid belonging to the quercetin group—an antioxidant of natural origin and a known component of food products [53].

DHQ is classified as a bioflavonoid, also known as vitamin P, which is not synthesized in the human body. Among all polyphenols, DHQ exhibits notably high antioxidant activity, along with anti-inflammatory and anti-allergic effects [54]. Table 2 presents selected technical characteristics of the DHQ used in this study.

In the food industry, DHQ is used as an additive to reduce oxidative degradation during storage and to extend product shelf life.

### 2.3. Polyethylene

Low-density polyethylene (LDPE), grade 15803-020, produced by SIBUR, was selected as the base polymer for film production. The main technical characteristics of the raw material are presented in Table 3.

This LDPE grade is used for the production of films and film-based products intended for food applications.

### 2.4. Modified Films

Film samples were produced using an SJ-28 laboratory-scale extruder. To improve dispersion in the polymer melt, a masterbatch was employed and mechanically blended with the base LDPE using a tumbling drum. The technical specifications are shown in Table 4.

The following polymer film compositions were prepared for the study: LDPE film based on grade 15803-20 (LDPE); LDPE film with 20.0 wt.% CaCO_3_ (LDPE 20 CaCO_3_); LDPE film with 40.0 wt.% CaCO_3_ (LDPE 40 CaCO_3_); LDPE film with 50.0 wt.% CaCO_3_ (LDPE 50 CaCO_3_); LDPE film with 70.0 wt.% CaCO_3_ (LDPE 70 CaCO_3_); LDPE film with 0.5 wt.% DHQ (LDPE + 0.5 DHQ); LDPE film with 20.0 wt.% CaCO_3_ and 0.5 wt.% DHQ (LDPE 20 CaCO_3_+ 0.5 DHQ); LDPE film with 40.0 wt.% CaCO_3_ and 0.5 wt.% DHQ (LDPE 40 CaCO_3_ + 0.5 DHQ); LDPE film with 20.0 wt.% CaCO_3_ and 1.0 wt.% DHQ (LDPE 20 CaCO_3_ + 1.0 DHQ); LDPE film with 40.0 wt.% CaCO_3_ and 1.0 wt.% DHQ (LDPE 40 CaCO_3_ + 1.0 DHQ).

The developed materials show strong potential for use in the food industry as functional packaging in various formats, with the ability to enhance product stability during storage.

### 2.5. Methods

The assessment of the level of antioxidant activity was carried out using the Tsvetyauza-01-AA (Russia, Moscow). The principle of operation chromatograph is based on the implementation of the high-performance liquid chromatography method in a non-isocratic or gradient mode. The analysis process is divided into two stages: separation of the sample into its constituent components; detection and measurement of the content of each component.

The components of the sample introduced into the chromatographic column move through the column at different speeds due to their different sorption capacities on the sorbent, and they reach the detector sequentially at different times.

The quantitative determination of each component is based on the analytical signal measured by the detector connected to the output of the chromatographic column. The principle of operation of the “TsvetYauza” 01-AA version is based on the implementation of flow-injection analysis. In flow-injection analysis, the sample is also introduced using a six-way valve, and a peristaltic pump (or other low-pressure pump) is used to supply the eluent. The concentration of the analyte is determined based on the signal from the amperometric detector.

Stress at break (σ), strain at break (ε), and the joint strength were measured in accordance to GOST 14236-81 [60] and GOST 12302-2013 [61] using a Shimadzu EZ-LX (Kyoto, Japan) tensile tester (with a 2 kN load cell and traverse stroke length 920 mm), equipped with TRAPEZIUM X software (ver. 1.5.2.).

The sessile drop method was used to determine the contact angle, defined as the angle formed between the tangent to the surface of a droplet of wetting liquid and the solid surface at the point of contact.

Surface morphology of the developed film samples was evaluated using scanning electron microscopy (SEM) on a Vega 3 scanning electron microscope (Tescan, Brno, Czech Republic), equipped with an energy-dispersive X-ray spectroscopy (EDS) detector, X-Act (Oxford Instruments, Abingdon, UK). Before imaging, the samples were sputter-coated with a ~20 nm platinum layer.

Fourier-transform infrared (FTIR) spectra of LDPE films with varying filler content were obtained using a Perkin Elmer Spectrum One FTIR spectrometer (Shelton, CT, USA) equipped with an attenuated total reflectance (ATR) accessory. For each sample, 64 scans were recorded at a resolution of 4 cm^−1^ in the range of 4000–650 cm^−1^.

Atomic force microscopy (AFM) was used to analyze the surface morphology of the films. Measurements were carried out in semi-contact mode using the Ntegra Prima scanning probe system (NT-MDT, Zelenograd, Russia), with configuration-specific settings including output signal amplitude, loop gain, piezo actuator frequency, and detector sensitivity. A “CSG01” cantilever (dimensions: 3.4 × 1.6 × 0.3 mm, tip radius 10 nm, spring constant 0.03 N/m) was used. The acquired data were processed and comparatively analyzed using the Nova software (ver.2.1.8.) package (NT-MDT), based on the INTEGRA (ver. 1.0.1) and Solver platforms (ver. 12.5).

Data visualization was performed using Microsoft Office applications (MS Word, MS Excel).

## 3. Results and Discussion

Experimental film samples based on LDPE grade 15803-20 with varying contents of CaCO_3_ and DHQ were produced. The width of the resulting film bubble was 175 ± 2 mm, and the film thickness was 35 ± 3 µm.

### 3.1. Results of Studies on the Antioxidant Activity of the Developed Films

**Figure 1:**
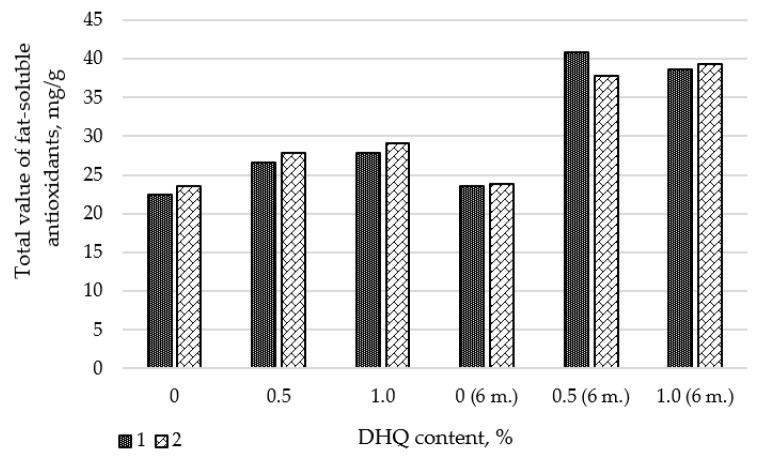
Changes in the AOA of polymer films modified with natural antimicrobial components at different concentrations: 1—LDPE 20 CaCO_3_; 2—LDPE 40 CaCO_3_.

As part of the work, studies were conducted to determine the change in antioxidant activity (AOA) of extracts from LDPE films with different levels of CaCO_3_ and DHQ. To conduct the experiment, we selected polymer film materials not only with different levels of the additive, but also after storing the products for 6 months. The results of the studies are presented in Figure 1. Since DHQ is almost insoluble in water, we used sunflower oil as a model medium for our research.

From the presented data it is seen that in samples with a concentration of DHQ 0.5 wt.% and 1.0 wt.% there is an increase in the total value of fat-soluble antioxidants (AOA). It should also be noted that a significant increase in AOA is observed in all samples containing DHQ during storage for 6 months. This may be due to the fact that during storage DHQ comes to the surface of the material and its concentration at the interface of the product-packaging increases. This effect is especially relevant for packaging products with long shelf life (in particular butter).

Visually, a small white coating is observed on the surface of the developed films during storage. We have proposed a working hypothesis that the introduced DHQ extract partially migrates to the surface of the films.

Extraction of DHQ into a model medium was performed by soaking 40 ± 2 g of the film in 100 mL of ethyl alcohol for 10 days. The resulting extract was then analyzed using liquid chromatography.

Based on the physical properties of DHQ, ethyl alcohol was chosen as a model medium for its quantitative determination. The results of determining the content of DHQ in alcohol extracts are presented in Table 5.

As can be seen from the data obtained, DHQ partially migrates to the surface of HDPE films with CaCO_3_, which confirms the hypothesis put forward.

To calculate the content of DHQ in the extracts, the areas of its peaks on the chromatograms of the test solution and the DHQ standard solution were measured. The conducted studies show that the use of high-performance liquid chromatography methods can be used for objective control of the content of DHQ in alcohol extracts from polymer materials modified with DHQ.

### 3.2. Results of Tensile Properties Evaluation

**Figure 2:**
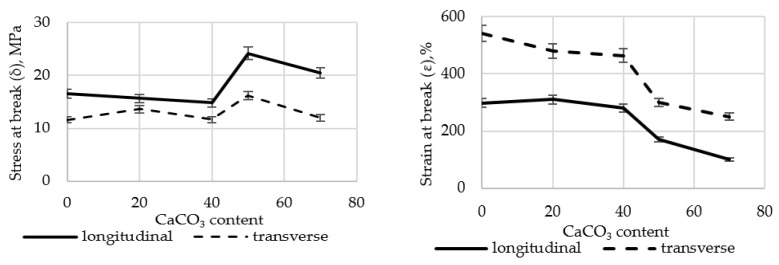
Changes in stress at break (σ) and strain at break (ε) in the longitudinal and transverse directions of LDPE films filled with CaCO_3_.

A comprehensive study was carried out to assess the effect of CaCO_3_ content on the tensile properties of polyethylene films. The results are presented in Figure 2. Tests of physical and mechanical characteristics were carried out in 20 consecutive measurements in the longitudinal and transverse directions. The average value was then calculated.

The results show that the σ (stress at break) of polyethylene films filled with up to 40 wt.% CaCO_3_ changes slightly. However, in samples containing more than 50.0 wt.% filler, stress at break increases by 45.9% in the longitudinal direction and by 39.8% in the transverse direction. Strain at break (ε) is more significantly affected. At 70 wt.% CaCO_3_ filler content, ε decreases by 66.4% (longitudinal) and 53.9% (transverse), respectively. This may be attributed to the fact that the addition of a low-molecular-weight inorganic filler alters the polymer matrix structure and weakens intermolecular interactions within the polymer.

CaCO_3_ is a rigid mineral filler that, when evenly distributed in a HDPE-based matrix, creates a kind of strong “skeleton.” Calcium CaCO_3_ particles redistribute mechanical loads in the polymer film, reduce local deformations, and increase tear resistance [62,63]. Introducing high concentrations of CaCO_3_ (>30–40 wt.%) leads to brittleness, as the bond between the particles and the polymer is disrupted [63,64]. Our findings correlate with ongoing research in this area.

**Figure 3:**
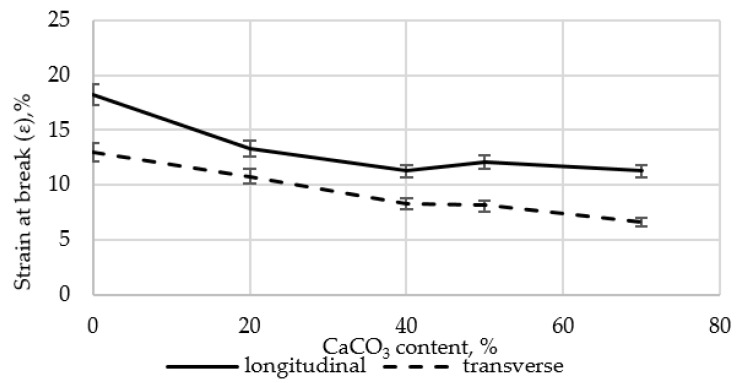
Changes in joint strength (σ) in the longitudinal and transverse directions of LDPE films filled with CaCO_3_.

The strength of joints in the polyethylene film with fillers was measured to determine their suitability for packaging applications. The results are presented in Figure 3.

Joint strength decreased by 38.3–49.8% in both longitudinal and transverse directions as CaCO_3_ content increased, compared to the LDPE film without fillers.

**Figure 4:**
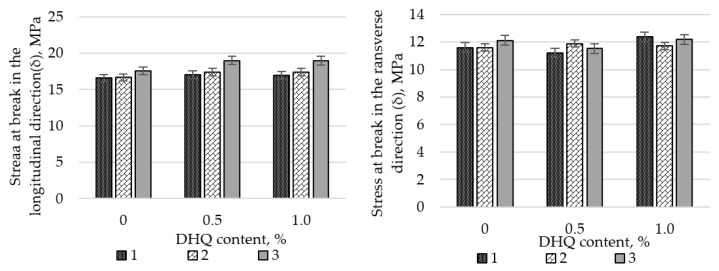
Changes in stress at break (σ) in longitudinal and transverse directions of LDPE films with varying CaCO_3_ and DHQ contents: 1—LDPE; 2—LDPE 20 CaCO_3_; 3—LDPE 40 CaCO_3_.

**Figure 5:**
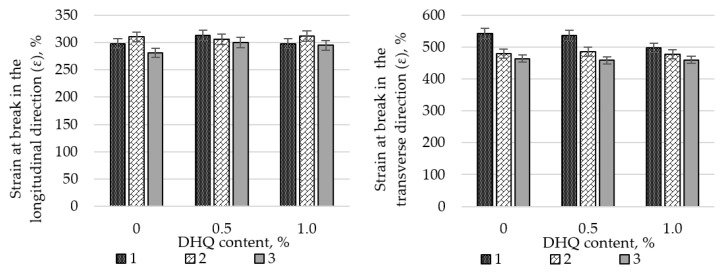
Changes in strain at break (σ) in the longitudinal and transverse directions of LDPE films with varying CaCO_3_ and DHQ contents: 1—LDPE; 2—LDPE 20 CaCO_3_; 3—LDPE 40 CaCO_3_.

Preliminary studies on the influence of CaCO_3_ on the tensile properties of LDPE films revealed a substantial decline in strength when the filler content exceeded 50 wt.%. Therefore, all subsequent experiments were performed using a CaCO_3_ content of no more than 40.0 wt.%. The results are shown in Figure 4 and Figure 5.

At the initial stage, the effects of CaCO_3_ and DHQ on the tensile properties of the developed LDPE-based films were investigated. The results indicate that the incorporation of CaCO_3_ by itself led to only minor changes in stress at break (σ). However, incorporation of DHQ at contents up to 1.0 wt.% into the polymer matrix containing various amounts of mineral filler resulted in an increase in the stress at break by up to 8.0% in the longitudinal direction and up to 6.5% in the transverse direction.

The strain at break (ε) of the samples also remained virtually unchanged, with deviations not exceeding 5.0% compared to the control film without fillers. Films with high filler contents exhibited higher values of stress at break than the unmodified LDPE base material. Depending on the filler content, stress at break values increased by 2.3% to 12.0% in the longitudinal direction and by 2.8% to 4.5% in the transverse direction.

### 3.3. Measurement of Water Contact Angle

**Figure 6:**
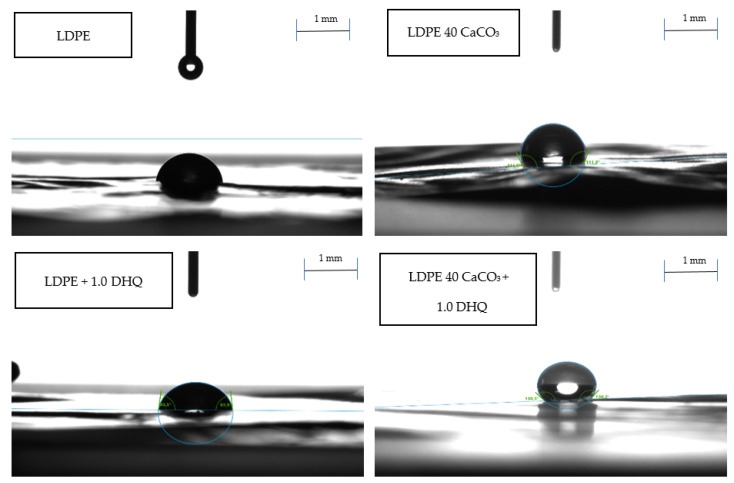
Contact angle at the surface of LDPE films modified with CaCO_3_ and DHQ.

The measurements were carried out in at least 15 consecutive trials. The contact angle (CA) was calculated as the average of the left and right angles of each droplet. The results of the experimental evaluation are presented in Table 6 and Figure 6.

As shown by the results, the contents of 0.5 wt.% and 1.0 wt.% DHQ does not significantly affect the adhesion properties of the LDPE film surfaces. A noticeable change in wettability is observed with the incorporation of CaCO_3_ into the polymer matrix. As illustrated in Figure 6, increasing the filler content to 40.0 wt.% leads to a more spherical droplet shape. The contact angle values vary within the following ranges: 84–86° for unmodified LDPE films, 90–100° for films with 20.0 wt.% CaCO_3_, and above 100° for films with 40.0 wt.% CaCO_3_. This effect may be attributed to the nature of the mineral filler and its distribution within the polymer matrix. From the literature data, it is known that not only the filler composition, but also its dispersion, as well as additionally introduced components, have a significant effect on the adhesive properties of the material surface [65,66]. For example, nano calcium carbonate has a strong effect on the surface wettability due to the large contact surface with water [65]. Contact angle, surface adhesion, and hydrophilicity of the modified polyethylene films must be taken into account when selecting appropriate applications. 

### 3.4. SEM Microstructural Analysis

**Figure 7:**
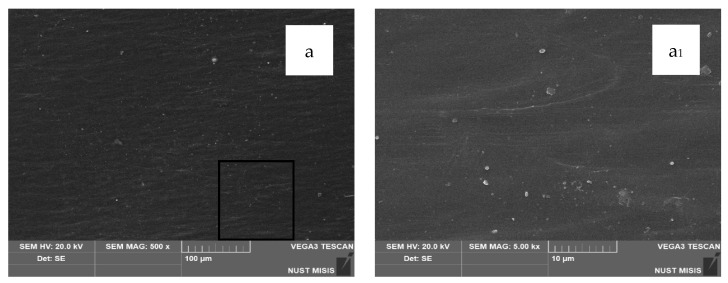

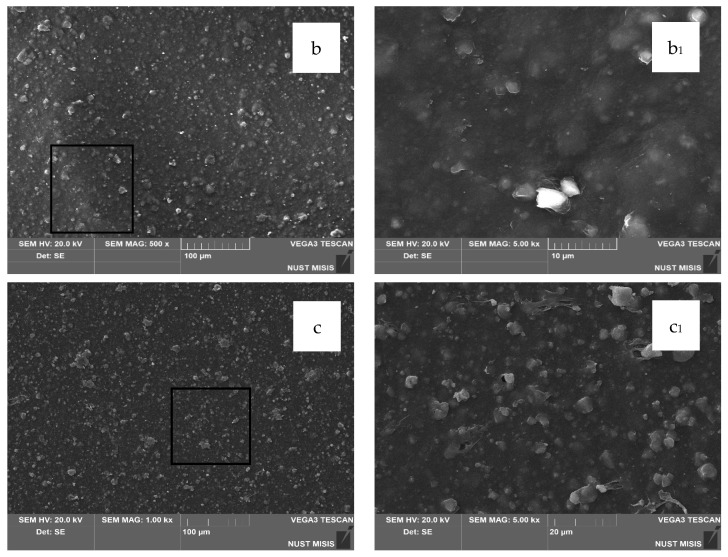
SEM images of film surfaces: (**a**,**a_1_**) LDPE; (**b**,**b_1_**) LDPE 20 CaCO_3_; (**c**,**c_1_**) LDPE 40 CaCO_3_.

Structural changes in the LDPE-based films modified with 20 wt.% and 40 wt.% CaCO_3_ were assessed using scanning electron microscopy (SEM). The results are presented in Figure 7. 

According to the SEM images, CaCO_3_ (20 wt.% and 40 wt.%) is distributed uniformly in the LDPE matrix, with no significant differences visible at 100 µm magnification. However, at 20 µm magnification, the surface of the film with 40.0 wt.% CaCO_3_ appears more porous, with numerous fine mineral particles observed near the surface. No through-holes, cracks, or tears were detected. At higher magnification, some CaCO_3_ particles appear to separate from the polymer matrix; however, they are not deep or extensive enough to influence the visual integrity of the film or interfere with subsequent evaluation of its physical-mechanical properties.

**Figure 8:**
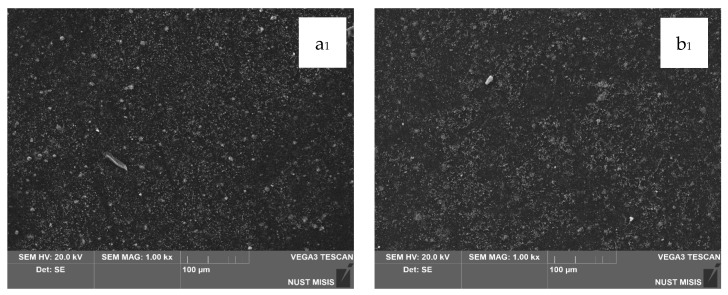

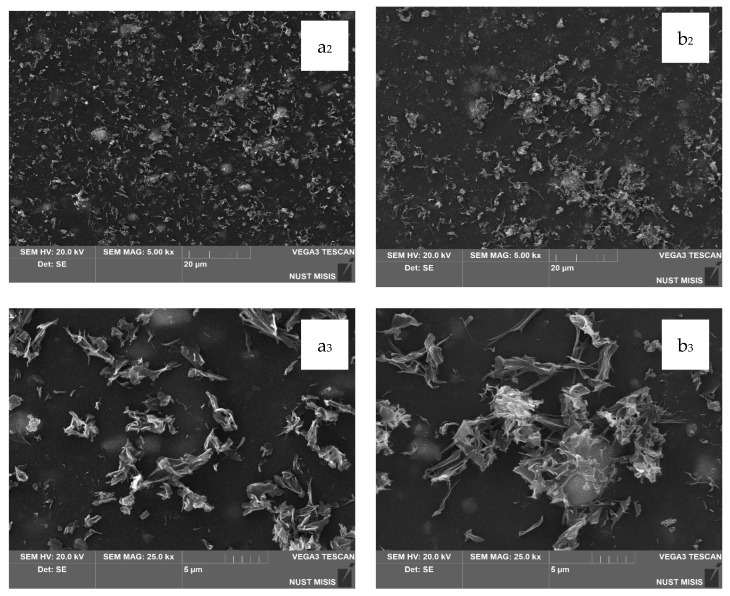
Comparative SEM images of film surfaces: (**a_1_**–**a_3_**) LDPE 20 CaCO_3_ + 0.5 DHQ; (**b_1_**–**b_3_**) LDPE 20 CaCO_3_ + 1.0 DHQ.

**Figure 9:**
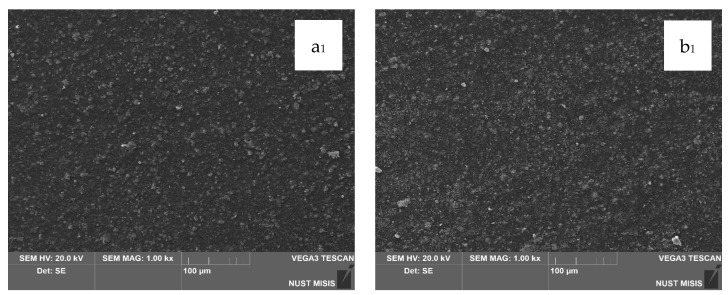

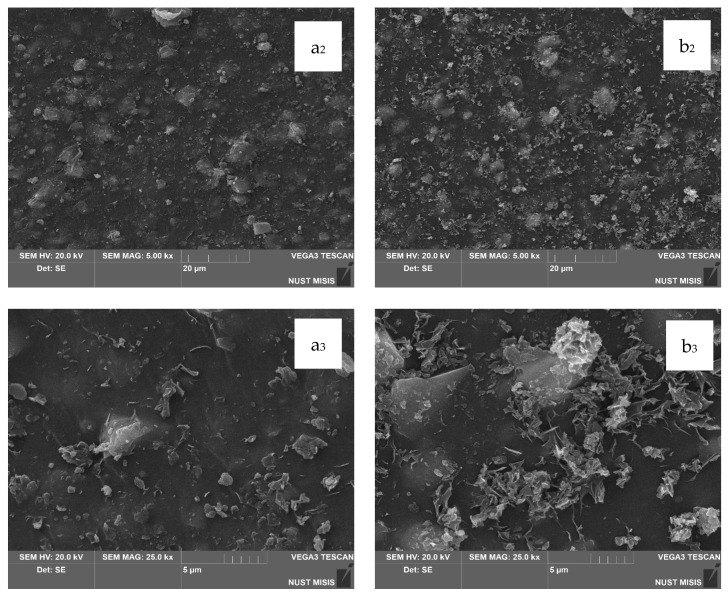
Comparative SEM images of film surfaces: (**a_1_**–**a_3_**) LDPE 40 CaCO_3_ + 0.5 DHQ; (**b_1_**–**b_3_**) LDPE 40 CaCO_3_ + 1.0 DHQ.

In the next stage, the surface morphology of LDPE films with 20.0 wt.% and 40.0 wt.% CaCO_3_ and 0.5 wt.% or 1.0 wt.% DHQ was assessed. The results are presented in Figure 8 and Figure 9.

The SEM images in Figure 8 show that the surfaces of LDPE films with 20.0 wt.% CaCO_3_ and 0.5 wt.% or 1.0 wt.% DHQ exhibit no visible chipping, tearing, or cracking. This indicates a sufficiently uniform dispersion of filler particles within the polymer melt. In comparison with films without DHQ, the modified samples show a notable presence of numerous fine crystalline particles on the surface. These are DHQ particles, which tend to migrate to the surface due to their relatively high molecular weight.

As shown in Figure 9, the surface morphology of LDPE films highly filled with 40.0 wt.% CaCO_3_ and 0.5 wt.% or 1.0 wt.% DHQ reveals no visible chipping, tearing, or cracking. In Figure 9b_1_–b_3_, with a 5 μm magnification, a large number of irregularly shaped particles with rather sharp edges are visible, which are not visually identifiable on the surface of the film without the addition of DHQ. In combination with the results of the quantitative detection of DHQ particles in the extracts using HPLC, we can confirm its presence on the surface of the developed materials. However, in contrast to the samples with 20.0 wt.% CaCO_3_, the number of fine antioxidant particles on the surface is noticeably lower. This may be due to the higher filler load, which could hinder the diffusion of DHQ molecules to the surface.

When comparing the micrographs in Figure 8 and Figure 9, it is noticeable that the base polymer base with a higher CaCO_3_ content appears denser, and on its surface, the number of DHQ particles is visually less than on the surface of the LDPE film with a CaCO_3_ content of 20.0 wt.%. This is most likely due to the density of the base material. Due to the high concentration of chalk, the diffusion of DHQ particles from the material to its surface is slowed down.

The microstructural analysis of films filled with an antioxidant confirms the presence of DHQ particles on the surface. The surface localization of DHQ particles may enhance the antioxidant activity precisely at the film–product interface in dairy applications. Additionally, DHQ migration occurs on both the inner and outer surfaces of the film, potentially improving adhesion properties. These surface observations are consistent with the results of the tensile properties analysis.

The incorporation of different fillers and modifiers can influence the degradation behavior of polymer materials, which in turn may affect their strength and contribute to microplastic formation and migration into packaged foods.

The structural analysis suggests that high CaCO_3_ content reduces surface homogeneity. However, no discrete agglomerates of filler or polymer were visually detected, indicating the potential morphological stability of the material during its use in food packaging applications.

### 3.5. AFM Analysis

**Figure 10:**
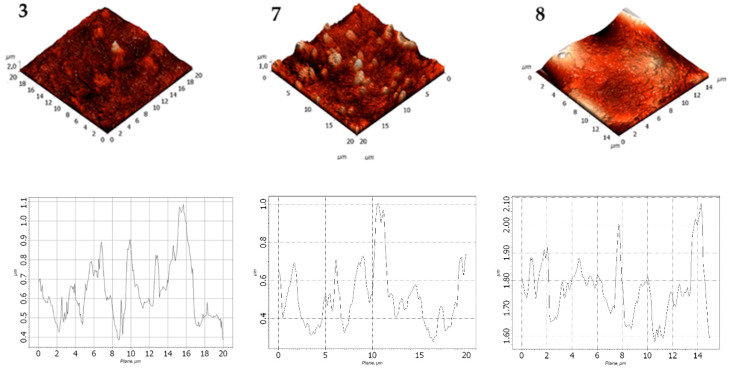
Surface morphology of films: 3—LDPE 40 CaCO_3_; 7—LDPE 40 CaCO_3_ + 0.5 DHQ; 8—LDPE 40 CaCO_3_ 1.0 DHQ.

**Figure 11:**
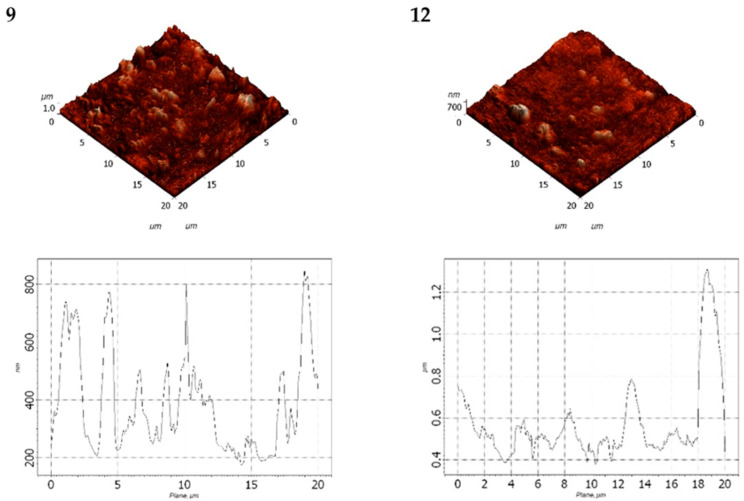
Surface morphology of films: 9—LDPE 20 CaCO_3_ + 0.5 DHQ; 12—LDPE 20 CaCO_3_ + 1.0 DHQ.

The obtained AFM data were processed and comparatively analyzed using the Nova SPM software package (NT-MDT, Zelenograd, Russia) based on the INTEGRA and Solver platforms. Surface images in various planes are presented in Figure 10 and Figure 11 and Table 7 and Table 8.

The statistical analysis of the surface topography of LDPE-based filled films is summarized in Table 7.

Based on the data in Table 7, it was observed that increasing the DHQ content in the formulation led to a decrease in surface roughness. However, the overall surface profile became more heterogeneous. For instance, the average surface roughness (Ra) of the LDPE film with 40 wt.% CaCO_3_ and 1.0 wt.% DHQ was 1.3 times lower than that of the LDPE film with 40 wt.% CaCO_3_.

It was found that decreasing the CaCO_3_ content to 20 wt.% while maintaining the same DHQ content (0.5 wt.% and 1.0 wt.% by weight) led to a significant reduction in both average surface roughness and the depth of surface pores (Table 8). 

The data obtained were consistent with those presented in Table 8: increase the DHQ content led to a decrease in Rvm and Rpm values. Specifically, the average surface roughness (Ra) decreased by a factor of 1.6.

The AFM analysis confirmed that CaCO_3_, when added at varying contents, influences both surface roughness and heterogeneity of the developed films. The incorporation of DHQ contributed to a smoother, more uniform surface morphology.

### 3.6. FTIR Analysis of Modified LDPE Films

LDPE is characterized by strong asymmetric (ν_as_ CH_2_) and symmetric (ν_s_ CH_2_) stretching vibrations observed at 2918 and 2851 cm^−1^, respectively. A medium-intensity absorption band at 1085 cm^−1^ corresponds to C–C stretching vibrations. The band near 1462 cm^−1^ is associated with asymmetric CH_2_ bending vibrations (δ CH_2_), which are typical for all polyethylene types. A weak band at 1373 cm^−1^ may be attributed to bending vibrations [67]. The absorption maximum at 714 cm^−1^ is also due to CH_2_ vibrations.

**Figure 12:**
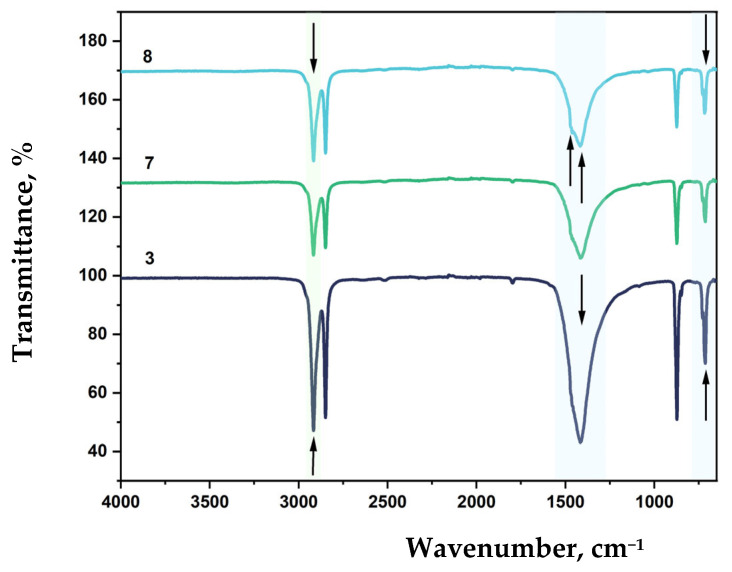
FTIR spectra, where: 3—LDPE 40 CaCO_3_; 7—LDPE 40 CaCO_3_ + 0.5 DHQ; 8—LDPE 40 CaCO_3_ + 1.0 DHQ.

**Figure 13:**
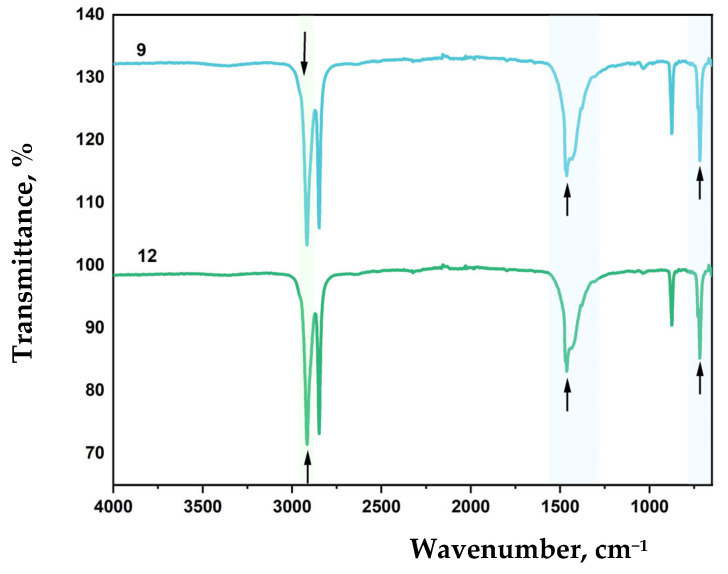
FTIR spectra of samples: 9—LDPE 20 CaCO_3_ + 0.5 DHQ; 12—LDPE 20 CaCO_3_ + 1.0 DHQ.

FTIR spectra of LDPE-based film samples containing CaCO_3_ and DHQ are shown in Figure 12 and Figure 13.

The effect of filler content on the structure of LDPE-based film materials produced by blown film extrusion was investigated using attenuated total reflectance Fourier-transform infrared (ATR-FTIR) spectroscopy. For films with up to 40 wt.% CaCO_3_, a slight shift in the absorption band corresponding to asymmetric stretching vibrations of –CH_2_– groups is observed, with the peak position moving to 2912 cm^−1^. This type of filler does not affect the RCH_2_–CO–CH_2_R group or the C–C bond. However, a new absorption peak appears at 873 cm^−1^, which is not typical for standard LDPE grades.

Further incorporation of 0.5 wt.% and 1.0 wt.% DHQ results in a reduction in the intensity of the asymmetric –CH_2_– stretching vibrations (ν_as_ CH_2_) and shifts this band to 2917 cm^−1^. With increasing DHQ content, a distinct shift of the CH_2_-related band from 714 to 718 cm^−1^ is also observed, along with the appearance of minor absorption bands at 1471 and 1455 cm^−1^, which can be due to C–H deformational vibrations and C=C valence vibrations of aromatic rings present in the DHQ [54,68].

A decrease in CaCO_3_ content to 20 wt.% while maintaining equivalent DHQ levels (0.5 wt.% and 1.0 wt.%) resulted in a shift of the asymmetric CH_2_ stretching vibration band (ν_as_ CH_2_) from 2912 cm^−1^ to 2916 cm^−1^ (Figure 12 and Figure 13). Additionally, lowering the crystalline filler content increased the intensity of bands at 1455, 1463, and 1472 cm^−1^.

As in previous samples, variation in filler content did not affect the C–C stretching band at 1085 cm^−1^.

This band of medium intensity consistently corresponds to C–C vibrations. The band at 1462 cm^−1^ reflects asymmetric CH_2_ bending vibrations typical of polyethylene [69,70].

The absorption maximum at 714 cm^−1^ also corresponds to CH_2_ vibrations. In highly crystalline polyethylene, this peak may split, leading to the appearance of an additional band at 730 cm^−1^.

## 4. Conclusions

Test samples of LDPE (grade 15803-20) films were produced and modified with 20.0 wt.%, 40.0 wt.%, 50.0 wt.%, and 70.0 wt.% CaCO_3_ and 0.5 wt.% and 1.0 wt.% DHQ (the natural antioxidant), as well as their combinations.

Tensile properties analysis revealed that incorporating 40.0 wt.% CaCO_3_ into LDPE had a negligible effect on the stress at break (σ) in both longitudinal and transverse directions compared to the unmodified control. Beyond this filler content, stress at break initially increased, then decreased as the filler content reached 70.0 wt.%. Changes in strain at break (ε) became pronounced at 40.0 wt.% filler content, with further increases leading to a loss of elasticity. These results suggest that an optimal filler content for maintaining tensile properties is no more than 40.0 wt.%.

It was demonstrated that adding up to 1.0 wt.% DHQ to the LDPE matrix with CaCO_3_ led to an increase in σ by up to 8.0% in the longitudinal direction and 6.5% in the transverse direction. Strain at break remained largely unchanged, with deviations of no more than 5.0% relative to the unmodified control film. Joint strength of LDPE films decreased by 18.0–60.0% as CaCO_3_ content increased (up to 40.0 wt.%), while the addition of DHQ up to 1.0 wt.% had a limited impact, with reductions not exceeding 6.0% compared to the control film.

Contact angle measurements confirmed that surface adhesion properties are significantly affected by the CaCO_3_ content. The contact angle ranged from 84–86° for unmodified LDPE, 90–100° for films with 20.0 wt.% CaCO_3_, and above 100° for those with 40.0 wt.% mineral filler.

SEM analysis of films with high CaCO_3_ content and DHQ revealed visible antioxidant particles on the film surface, suggesting enhanced antioxidant potential at the interface between the film and dairy products. DHQ was found to migrate to both the outer and inner surfaces, which may also influence the adhesion behavior of the material.

AFM analysis confirmed that CaCO_3_ 40.0 wt.% content alters the surface roughness and heterogeneity of the films. The presence of DHQ contributed to surface smoothing and improved homogeneity.

FTIR spectroscopy revealed that the incorporation of CaCO_3_ influenced the overall spectral profile of polyethylene, resulting in decreased peak intensities depending on the concentration of the filler. For LDPE 40 CaCO_3_ films, there is a slight shift of the absorption band maximum corresponding to the asymmetric valence vibrations of CH_2_ to 2912 cm^−1^. CaCO_3_ does not affect the RCH_2_-CO-CH_2_R group and the C-C bond, but a new peak appears at 873 cm^−1^, which is not typical for some LDPE grades.

## Figures and Tables

**Table 1:** Technical specifications of the CaCO_3_ masterbatch used.

Characteristic	Value
Appearance	White granules
Granule size, mm	3–4
Whiteness, %	>97
Bulk density, g/cm^3^	1.9
MFR (190 °C), g/10 min	3
CaCO_3_ content, %	80.0
Base polymer content (LDPE), %	20.0
Mean particle size of CaCO_3_, µm	2.0
Moisture content, %	<0.15

**Table 2:** Technical characteristics of DHQ.

Parameter	Description
Compound name	Dihydroquercetin
Raw material	Dahurian larch *Latix gmelinii* (Rupr.)
Plant part used	Root wood
Country of origin	Russia
Substance class	Bioflavonoids
Composition	Dihydroquercetin > 90%
Other flavonoids	Aromadendrin, eriodictyol, quercetin, taxifolin, pinocembrin, total < 10%
Appearance	Fine powder
Color	White to pale yellow
Solubility	Soluble in ethanol, hydroalcoholic solutions, ethyl acetate; slightly soluble in water; insoluble in chloroform, ether, and benzene
Moisture content	<2%

**Table 3:** Main characteristics of LDPE.

Characteristics	Method	Value
Flow index of melt	GOST 11645 [55]	2.0 g/10 min
Density	GOST 15139 [56]	0.92 g/cm^3^
Stress at yield	GOST 11262 [57]	9.3 MPa
Stress at break	GOST 11262 [57]	11.3 MPa
Extractable substances content	GOST 26393 [58]	0.4%
Odor intensity from aqueous solution	GOST 22648 [59]	Not more than 1.0 point

**Table 4:** Main characteristics of the technical specifications of the laboratory extruder.

Characteristic	Value
Screw diameter	28 mm
Maximum film bubble width	200 mm
Die (film die) diameter	40 mm
Film thickness	0.010–0.050
Length-to-diameter ratio	34:1
Number of temperature control zones	4
Extruder zone temperatures:	
Zone 1	145 °C
Zone 2	150 °C
Zone 3	153 °C
Zone 4	157 °C

**Table 5:** Content of DHQ in alcohol extracts from modified polymer materials.

Name of the Sample	Sample Weight, g	wt.% DHQ in the Film	Content DHQ in the Sample, g	Content DHQ in the Hood, g
LDPE film 20 wt.% CaCO_3_ and DHQ	40.8	0.5	0.0971	0.0091 ± 0.0004
LDPE film 20 wt.% CaCO_3_ and DHQ	41.6	1.0	0.2135	0.0183 ± 0.0004
LDPE film 40 wt.% CaCO_3_ and DHQ	40.5	0.5	0.096	0.0089 ± 0.0004
LDPE film 40 wt.% CaCO_3_ and DHQ	41.7	1.0	0.216	0.0196 ± 0.0005

**Table 6:** Results of experimental assessment of the water contact angle on the surface of LDPE films modified with CaCO_3_ and DHQ.

Indicator	LDPE Film with Varying CaCO_3_ and DHQ Contents **								
	№1	№2	№3	№4	№5	№6	№7	№8	№9
Mean CA (avg) [°] *	85.35	82	84.34	89.54	90.76	86.07	107.97	114.4	129.84
Mean CA (l) [°] *	85.57	81.59	84.13	89.55	90.62	85.82	107.86	114.4	129.93
Mean CA (r) [°] *	85.13	82.41	84.55	89.53	90.91	86.33	108.08	114.4	129.75
Mean triple-phase point (l) [mm] *	7.5	7.7	7.6	7.8	7.6	7.6	7.6	9.1	8.8
Mean triple-phase point (r) [mm] *	11	11.5	11.4	11.2	11.4	11.3	10.8	12.2	11.2
Mean diameter [mm]	3.52	3.8	3.83	3.42	3.78	3.75	3.17	3.1	2.39
Mean volume [µL]	9.63	10.99	11.90	9.86	13.86	11.88	13.56	14.75	12.16

* Mean CA (avg)—the average value of the left and right contact angles. Mean CA (l)—the average value of the left angle. Mean CA (r)—the average value of the right angle. Triple-phase point—the point at which the solid, liquid, and vapor phases are in contact. Mean triple-phase point (l)—average value of the left three-phase points. Mean triple-phase point (r)—average value of the right three-phase points. ** №1—LDPE; №2—LDPE + 0.5 DHQ; №3—LDPE + 1.0 DHQ; №4—LDPE 20 CaCO_3_; №5—LDPE 20 CaCO_3_ + 0.5 DHQ; №6—LDPE 20 CaCO_3_ + 1.0DHQ; №7—LDPE 40 CaCO_3_; №8—LDPE 40 CaCO_3_ + 0.5 DHQ; №9—LDPE 40 CaCO_3_ + 1.0 DHQ.

**Table 7:** Statistical parameters of surface roughness for the films.

Parameter	Sample Number		
	3	7	8
Average roughness (Ra), µm	0.178	0.165	0.133
Average maximum depth of surface pores (Rvm), µm	0.517	0.446	0.659
Average maximum peak height of roughness (Rpm), µm	1.728	1.003	1.033

**Table 8:** Statistical parameters of surface morphology for samples with reduced CaCO_3_ content.

Parameter	Sample Number	
	9	12
Average roughness (Ra), µm	0.122	0.077
Average maximum depth of the roughness valley (Rvm), µm	0.326	0.251
Average maximum peak height of roughness (Rpm), µm	0.919	0.633

## Data Availability

Data are contained within the article.
